# COVID-19: lipid disruption is pushing the envelope

**DOI:** 10.1016/j.jlr.2022.100240

**Published:** 2022-06-10

**Authors:** Garret A. FitzGerald

**Affiliations:** Institute for Translational Medicine and Therapeutics, Perelman School of Medicine, University of Pennsylvania, Philadelphia, PA, USA

**Keywords:** aPL, aminoPhospholipid, COVID-19, coronavirus disease 2019, PE, phosphatidylethanolamine, SARS-CoV-2, severe acute respiratory syndrome coronavirus 2

A plethora of articles have been published on severe acute respiratory syndrome coronavirus 2 (SARS-CoV-2), and science has delivered, given the rapid development of vaccines and of novel antiviral therapeutics evaluated for their efficacy efficiently in platform trials. An unfolding story of interferon genetics and autoantibodies has begun to help us parse the reasons for varied susceptibility to severe disease and sequencing, and tracking at a global level has allowed for the rapid detection of new variants as they emerge. For all that progress and the political allocation of substantial resource to the World Health Organization in support of future pandemic preparedness, we failed to deploy the social science that would have anticipated what is politely called “vaccine hesitancy” or the psychological strategies to cope with what remains a continuing problem. This phenomenon and the failure equitably to distribute the opportunity for vaccination and novel therapeutics at a global level represent the biggest challenges to concluding the pandemic of coronavirus disease 2019 (COVID-19).

In the *Journal of Lipid Research*, Saud *et al.* (1) presented a therapeutic opportunity that involves neither vaccines nor drugs. It may translate into a cheap and effective approach of relevance, not just to COVID-19, but generally to viral pandemics of the future; swilling and gargling mouthwash to disrupt the integrity of the lipid envelope that facilitates viral infection and proliferation.

Attention has been payed to the modulation of cellular entry afforded by linoleic acid binding to SARS-CoV-2 (2), to how the lipid composition of cell membranes can influence viral entry by mediating fusion or affecting receptor conformation, and how, upon infection, viruses can reprogram cellular metabolism to remodel lipid membranes and fuel the production of new virions (3). In addition, eicosanoids and sphingolipids have been implicated in the immune response evoked by the virus, which, when unrestrained, contributes substantially to the clinical phenotype of severe disease (4). While there have been articles describing the lipidomic response to SARS-CoV-2 infection (5, 6), these have been relatively few when compared with interrogation of the immunophenotype (7, 8). How the high- and low-abundance lipids that are formed in response to infection integrate in a multiomic context to drive the immune response remains to be described.

The novel contribution from Saud *et al.* (1) is a focus on the viral envelope. Because coronaviruses bud from the endoplasmic reticulum–Golgi intermediate complex and exit via lysosomal secretion, the lipid composition of their envelope might be expected to differ from that of cell membranes. Indeed, that is the case, perhaps in a way that affords therapeutic opportunity.

Viral lipids were extracted following infection of both VeroE6 and A459 cells and analyzed using both targeted and untargeted LC/MS/MS approaches. VeroE6 cells were originally isolated from kidney epithelial cells extracted from an African green monkey, and A549 cells were isolated from the lung tissue of a 58-year-old Caucasian male with lung cancer.

Across the two cell types, roughly 260 lipids were reproducibly detected. The most abundant were phosphatidylcholine, phosphatidylethanolamine (PE), and phosphatidylinositol, along with several respective lyso, and ether/plasmalogen forms, and the relative proportions differed somewhat across the two preparations. The most abundant fatty acids detected were 16:0, 18:0, and 18:1, whereas others again differed, depending on the cell type infected. The authors focus on the high content of aminoPhospholipids (aPLs) in the viral envelope and specifically on the very high percentage of phosphatidylserine and PE that is externalized (although this again differed somewhat between the cells infected) compared with mammalian cell membranes. The authors highlight the comparatively small degree of phosphatidylserine/PE externalized on the membranes of activated platelets, where they are sufficient to catalyze and support the assembly of the prothrombinase complex (9). Hence, they speculate a role for externalized aPLs in the viral envelope driving the thrombotic complications of severe COVID-19. Consistent with this, they show that virions dose dependently shorten the activated partial thromboplastin time, a measure of induced coagulation in vitro. Infection of endothelial cells with SARS-CoV-2 as such would afford a potential site of such a thrombogenic mechanism (10).

Having implicated the viral envelope as a potential contributor to one aspect of disease severity, they report surfactant (such as cetylpyridinium chloride)-dependent differential toxicity and efficacy amongst seven oral rinses to suppress viral infectivity in VeroE6 cells in the presence of a soil load to mimic the components of the nasal/oral cavity. Finally, they attempt to assess the ability and persistence of several rinses to suppress oral viral load in patients infected with SARS-CoV-2 in a randomized trial. Unfortunately, their trial design was undermined by a rapid decline in cases during their study such that they could only gather preliminary information. However, this was encouraging and provided data consistent with what had been seen in vitro, suggestive of a differential efficacy of this approach, at the least sufficient to permit safe oral examination.

The authors are to be applauded for bringing delayed attention to the lipid composition of the viral envelope. Their suggestion of a prothrombotic contribution from its disproportionate content of exposed aPLs is provocative although many factors of relevance—blood flow, relative abundance compared with activated platelets in a developing thrombus, and perhaps most importantly, the modulatory effect of the concomitant inflammatory response on envelope lipids as viruses infect native epithelial cells of the respiratory tract—remain to be addressed. Thrombotic events complicate severe COVID-19, but no more so than in other severe viral illnesses (11), and low-dose aspirin does not influence the clinical course when begun early after likelihood of hospitalization (12). Rinsing and gargling may reduce viral load in the oropharynx, but infection in the respiratory tree already established before this intervention will be unaltered.

If these rinses can be shown safely to reduce oropharyngeal viral load in a suitably powered clinical trial, this may prove to be a simple and perhaps broadly acceptable approach to attenuating the consequences of infection, not only of SARS-CoV-2 but also of other viruses. Most importantly, we would then have a therapeutic intervention, perhaps acceptable to the “vaccine hesitant” but certainly one that is economically practical to make available across the world.

## Conflict of interest

The author declares no conflicts of interest with the contents of this article.
